# 17beta-estradiol induced vitellogenesis is inhibited by cortisol at the post-transcriptional level in Arctic char (*Salvelinus alpinus*)

**DOI:** 10.1186/1477-7827-2-62

**Published:** 2004-09-02

**Authors:** Hakan Berg, Carina Modig, Per-Erik Olsson

**Affiliations:** 1 Department of Molecular Biology, Umea University, Umea, Sweden; 2 Department of Marine Science, University of Texas Marine Science Institute, University of Texas, Port Aransas, Texas, USA; 3 Department of Natural Science, Unit of Molecular Biology, Orebro University, Orebro, Sweden

## Abstract

This study was performed to investigate stress effects on the synthesis of egg yolk precursor, vitellogenin (Vtg) in Arctic char (*Salvelinus alpinus*). In particular the effect of cortisol (F) was determined since this stress hormone has been suggested to interfere with vitellogenesis and is upregulated during sexual maturation in teleosts. Arctic char Vtg was purified and polyclonal antibodies were produced in order to develop tools to study regulation of vitellogenesis. The Vtg antibodies were used to develop an enzyme-linked immunosorbent assay. The corresponding Vtg cDNA was cloned from a hepatic cDNA library in order to obtain DNA probes to measure Vtg mRNA expression. Analysis of plasma from juvenile Arctic char, of both sexes, exposed to different steroids showed that production of Vtg was induced in a dose dependent fashion by 17β-estradiol (E2), estrone and estriol. Apart from estrogens a high dose of F also upregulated Vtg. In addition, F, progesterone (P) and tamoxifen were tested to determine these compounds ability to modulate E2 induced Vtg synthesis at both the mRNA and protein level. Tamoxifen was found to inhibit E2 induced Vtg mRNA and protein upregulation. P did not alter the Vtg induction while F reduced the Vtg protein levels without affecting the Vtg mRNA levels. Furthermore the inhibition of Vtg protein was found to be dose dependent. Thus, the inhibitory effect of F on Vtg appears to be mediated at the post-transcriptional level.

## Introduction

The major proteinaceous egg yolk precursor vitellogenin (Vtg) is a large complex lipoglycophosphoprotein produced under estrogenic control in the liver of sexually maturing female oviparous animals. The estrogenic control of Vtg is mediated by binding of the most potent estrogen, 17-β-estradiol (E2), to the hepatic estrogen receptor (ER) [1]. The ER-E2 complex activates the transcription of the Vtg-genes by binding to estrogen responsive elements [1]. Vtg is transported from the liver as a dimer via the circulation to the oocytes, where it is taken up by receptor mediated endocytosis [2,3] and proteolytically cleaved into the smaller yolk units lipovitelin, phosvitin [4,5] and phosvettes [6], which serve as a nutritional source for the growing embryos [7]. Studies have shown that Vtg bind metal-ions such as zinc, calcium [8,9] and magnesium [10]. It has been suggested that Vtg is involved in the transport of metal-ions, crucial for embryonic development, into the growing oocyte [11].

A number of Vtg genes have been characterized in a wide variety of oviparous species and it has been shown that the Vtg genes are highly conserved [12,13]. The Vtg-genes belong to a small gene family where the number of genes varies depending on species [7,14,15]. The different genes give rise to multiple forms of the protein, which are expressed at different times during oogenesis. This indicates that Vtg isoforms may have different roles during oocyte maturation and embryonic development [5]. Vitellogenin genes are present in both females and males but the lack of estrogens in the males prevents the expression of the protein under normal conditions [16].

In teleosts, cortisol (F) is released from interrenal cells in response to stress. It has been shown that F affects reproduction by decreasing the amount of gonadotropins produced by the pituitary, the amount steroids present in the plasma and by reducing gamete quality [17]. Earlier studies on stress responses on teleost reproduction are ambiguous. In some studies F does not interact with E2 systems [16,18], while other studies indicate that F interferes with the binding of E2 to ER, thereby decreasing hepatic Vtg production [19]. It has been proposed that this ambiguity is due to species-specific responses to F thereby giving rise to different stress responses in different species.

Many manmade substances with endocrine disrupting properties (EDS) are present in the environment. It has been observed that stress responses are induced in organisms when exposed to EDS. Numerous EDS have been shown to impair reproductive function in teleost fish [18]. It is therefore important to examine how stress responses interfere with the expression of commonly used biomarkers. Exposure of male or juvenile fish to estrogenic substances results in stimulation of Vtg production [20,21]. Vtg is therefore widely used as a biomarker for estrogenicity [22,23]. In the present study Arctic char Vtg was characterized and the effect of F on E2 induced vitellogenesis was investigated.

## Materials and methods

### Experimental animals and rearing conditions

Juvenile Arctic char with an average weight of 18.4 ± 10.7 g were obtained from the National Swedish Board of Fisheries Research Station, Kälarne, Sweden. They were kept in indoor 50 l tanks with a continuous flow-through water system with temperature and photoperiods as close to the natural conditions as possible. The fish were allowed to acclimatize for 1 week prior to initiating the experiments. No food was administered to the fish during the experiments.

### Fish treatment and sampling

Vtg synthesis was induced by intraperitoneally (*i.p*.) injection of Arctic char with 10^-6 ^M E2. Peanut oil was used, as a carrier and control injections were made with carrier alone. The fish were kept for four days prior to sampling. Plasma was collected by centifugation and used to purify Vtg in order to develop polyclonal antibodies.

Juvenile Arctic char were injected *i.p*. with different doses of E2, estriol and estrone (end-concentrations ranging between 10^-9 ^to 10^-6 ^M) and F, corticosterone, cortisone, 11-ketotestosterone and progesterone (P) (end-concentrations ranging between 10^-8 ^to 10^-5 ^M) to determine the effect of these 8 hormones on Vtg expression. Four days after injection the fish were sacrificed, bled and the livers were removed. The obtained blood was centrifuged at 5000 × g for 1 minute in order to separate the blood cells from the plasma. The plasma and livers were immediately frozen in liquid nitrogen and stored at -80°C until analyzed.

To further investigate the effects of steroids on Vtg production, different doses of E2 (end-concentration ranging between 10^-8 ^to 10^-6 ^M) was administered *i.p*. with or without co-injection of F (end-concentration ranging between 10^-8 ^to 10^-4 ^M), P (10^-5 ^M) or tamoxifen (Tam) (10^-5 ^M). After four days the fish were sacrificed, the liver and plasma were collected and stored as described above.

### Hormone determinations

E2 and F plasma levels were determined by radioimmunoassay according to manufacturers instructions (E2-Coat-a-Count, DPC, USA, F-Spectria Cortisol RIA, Orion Diagnostica, Espoo, Finland). The measurements were made in triplicates.

### Isolation of vitellogenin

Prior to chromatography, the Vtg in the plasma was concentrated by selective precipitation as described by [24]. 0.5 ml of plasma were mixed with 2 ml of 20 mM EDTA, and precipitation was obtained by subsequently adding 0.1 ml 0.5 M MgCl_2_. The precipitate was collected by centrifugation at 5000 × g for 15 minutes at +4°C, and the supernatant was discarded. The obtained precipitate was re-dissolved in 1 ml of 1 M NaCl prior to a second precipitation, performed by lowering the ionic strength of the sample by adding 10 ml of ultrapure deionized water (MQ). The precipitate was collected by centrifugation at 5000 g for 15 minutes +4°C and the pellet was dissolved in 1 ml of 1 M NaCl prior to fast performance liquid chromatography (FPLC).

All solutions used for FPLC contained aprotinin (0.5% v/v) and were filtered through 0.22 μm filters and degassed. The column used was a Resource Q (Pharmacia, Sweden), which was equilibrated with five volumes of 20 mM Tris-HCl pH 8.0 (Buffer A). The plasma was diluted 50 times and 0.5 ml of the diluted sample was loaded onto the equilibrated column. Unbound plasma-proteins were eluted with 5 ml of buffer A. The bound proteins were separated by a 15 ml linear gradient from 0.00 M to 0.50 M NaCl. The column was washed with 5 ml of 1.0 M NaCl to ensure that no other proteins remained bound. The flow-rate was 1 ml min^-1 ^and 1 ml fractions were collected. The obtained Vtg was stored in 50% (v/v) glycerol until further analysis.

In order to control the efficiency of the different purification steps, 10 μg of total protein from each of the steps were run onto an 8% discontinuous polyacrylamide gel (SDS-PAGE) and stained with Coomassie brilliant blue. The FPLC purified Vtg was used to immunize rabbits (AgriSera, Vindeln, Sweden).

### Western blot analysis

To identify Vtg present in the plasma of sampled fish total protein was loaded onto a discontinuous polyacrylamide gel with a 2 or 4% stacking gel and an 8% separating gel [25]. Following electrophoresis, the proteins were blotted onto nitrocellulose membrane (Hybond-ECL™) or PVDF membrane (Amersham) using either semi-dry or tank transfer system (Bio-Rad Laboratories). To block non-specific antibody binding, the membranes were incubated with fat-free milk powder (5% in Tris-buffered saline, pH 7.4, containing 0.5% Tween 20; TBS-T). The membranes were incubated with primary antibody for 1 hour at room temperature (RT) or over night at 4°C. The primary antibodies were directed against Arctic char Vtg and diluted 1:5000 in TBS-T. The membranes were washed 3 × 5 minutes in TBS-T and incubated for 1 hour with the secondary antibody (Horseradish Peroxidase-conjugated anti-rabbit Ig, DAKO A/S Denmark), diluted 1:5000 in TBS-T. Prior to detection, the membranes were washed 3 × 5 minutes in TBS-T. The detection was performed using the ECL™ detection system (Amersham Pharmacia Biotech, Uppsala, Sweden)

### Two-dimensional polyacrylamide gel electrophoresis analysis

Two-dimensional poloyacrylamide gel electrophoresis (2D-PAGE) of plasma proteins was run on Multiphor II electrophoretic unit (Pharmacia Biotech) according to the manufactures manual. Separation in the first dimension (IEF) was performed using linear pH 4–7 gradient immobiline DryStrips (Amersham Biosciences), 40μg protein was loaded per strip. In the second dimension an 8–18% gradient polyacrylamide gel (ExcelGel SDS, Amersham Biosciences) was used. The gels were either stained with Coomassie Brilliant Blue or the proteins was transferred to PVDF-membrane. The blot was blocked with fat-free milk powder (5%) in TBS, pH 7.4, followed by anti-Vtg (diluted 1:3000) incubation over night at 4°C. After 3 × 10 minutes washes in TBS-T the membrane was incubated for 2 hours with the secondary antibody (HRP-conjugated anti-rabbit Ig, Amersham Biosciences), diluted 1:3000. Prior to detection, the membranes were washed 2 × 15 minutes in TBS-T and 1 × 5 minutes in TBS. For detection of antibody staining ECL™ reagents was used and the chemiluminescent signal was detected on Hyperfilm MP (Amersham Biosciences).

### ELISA procedure

Quantification of plasma Vtg was performed by enzyme-linked immunosorbent assay (ELISA), prepared by coating 96 well microtiter plates (Nunc A/S, Roskilde, Denmark) with plasma-samples diluted in coating buffer (0.1 M Na_2_CO_3_, pH 9.6). A standard curve made from purified Arctic char Vtg was also loaded onto each plate as a control. The plates were incubated at RT for 1 hour prior to blocking non-specific binding by adding phosphate buffered saline, pH 7.6 (PBS) containing 1% dry milk to each well. The plates were washed in PBS containing 0.05% Tween 20 (PBS-T) before addition of primary antibody. The polyclonal primary antibodies against Arctic char Vtg were diluted 1:10000 in PBS-T, added to the plates and incubated in RT for 1 hour. After washing the plates with PBS-T, a secondary antibody incubation was performed by adding HRP-conjugated goat-antirabbit polyclonal antibodies (DAKO A/S Denmark) diluted 1:5000 in PBS-T. The plates were incubated for 1 hour at RT prior to PBS-T-wash and detection. The detection was performed using a peroxidase substrate kit (Horseradish peroxidase substrate kit, BIO-RAD, Hercules, CA, USA). The plates were read at 415 nm, using a microplate reader (BIO-RAD microplate reader Model 550). All samples were analyzed in triplicates. To establish the titer of the polyclonal Vtg antibodies an ELISA with the wells loaded with equal amount VTG and various antibody concentrations were used. The detection limit of the ELISA procedure was determined by loading a standard curve of pure Vtg and using a fixed antibody concentration.

### cDNA cloning

A ZAP Express cDNA library (Stratagene, La Jolla, CA, USA) from E2 induced Arctic char liver was used. The library was screened using a probe constructed from the rainbow trout pSG Vg 5.09 cDNA clone [26]. The isolated phage DNA clones were subjected to *in vivo *excision prior to sequencing. Positive clones from the screening were selected for sequencing by dot blot and Northern blot analysis (data not shown) and sequencing was performed using Thermo Sequenase (Amersham).

### RNA extraction and slot blot procedure

Total RNA was isolated from Arctic char livers according to Chomczynski and Sacci [27]. Slot blot analysis was used to quantify Vtg mRNA levels. Nylon membranes (Hybond N, Amersham) were soaked in 20 × SSC (1 × SSC, 0.15 M NaCl; 15 mM sodium citrate buffer, pH 7.0). RNA samples were prepared by mixing 10 μg of total RNA with 6 × SSC and 7.5% formaldehyde and heating to 68°C for 15 min. The RNA samples were immediately cooled down on ice prior application onto the slot blot. Following slot blot the membranes were washed twice with 2 × SSC and cross-linked on both sides before hybridization against a single stranded digoxigenin (DIG) labeled cRNA Arctic char Vtg probe. Hybridization and detection of Vtg was performed as described previously [28]. Quantification of the mRNA was performed with Quantity One version 4.2.3 (BIO-RAD Laboratories AB, Sundbyberg, Sweden). In order to normalize the amount of total RNA in each slot, a slot blot membrane was hybridized with a DIG-labeled probe complementary to Arctic char 18S rRNA The probe was made as follow: total RNA from liver was used for first-strand cDNA synthesis according to the manual of Amersham. 18S fragments was PCR amplified by 30 cycles of 94°C for 30 seconds, 57°C for 30 seconds and 72°C for 30 seconds, using Quantum RNA classic 18S PCR primer pair (Ambion). The PCR fragment was cloned into pGEM-T vector (Promega). The purified plasmid was used as DNA-template in a PCR reaction (as above) to synthesize the DIG-labeled 18S DNA probe (DIG-11-dUTP was obtained from Roche). The Vtg mRNA levels in liver from control fish was arbitrarily set to 1.

### Statistics

Significance was calculated using one-way ANOVA followed by Bonferroni's multiple comparison test with a P < 0.05. All statistical analysis was performed using GraphPad Prism version 3.02 for Windows (GraphPad Software, San Diego California USA).

## Results

**Figure 1:**
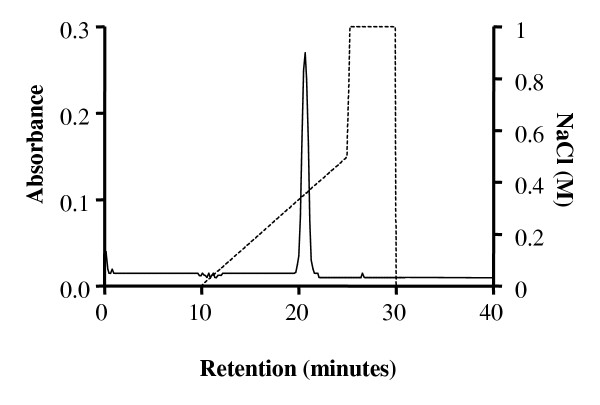
Elution profiles from Resource Q-chromatography of E2 treated Arctic char plasma proteins following selective precipitation. The linear gradient used was between 0.00–0.50 M NaCl. The absorbance was measured at 280 nm. The pure Vtg gave rise to one homogenous absorbance peak at an ion concentration of 0.37 M.

Administration of E2 to juvenile Arctic char led to a rapid increase in plasma protein concentrations from 6.2 ± 0.3 mg/ml in control fish to 21.4 ± 0.5 mg/ml in E2 injected fish. The plasma contained low molecular weight proteins that were excluded from the preparation by sequential precipitations. The final pellet was re-dissolved in 1 M NaCl and subjected to FPLC purification. A single absorbance peak containing Vtg was identified at an ion concentration of 0.37 M (Fig. 1). This peak was not present in plasma from untreated juvenile fish (data not shown). SDS-PAGE analysis showed that the purified Vtg had a molecular mass of 185 kDa.

**Figure 2:**
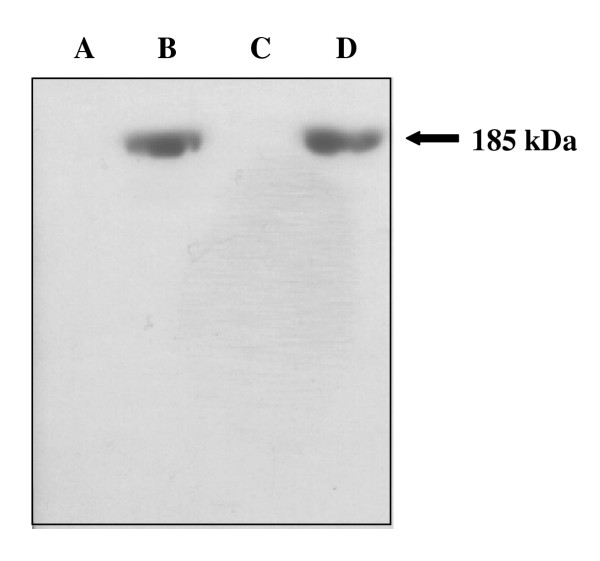
Western blot analyses using a polyclonal antibody against Arctic char Vtg, on plasma from **A) **untreated juveniles. **B) **E2 exposed juveniles. **C) **male fish. **D) **female fish.

**Figure 3:**
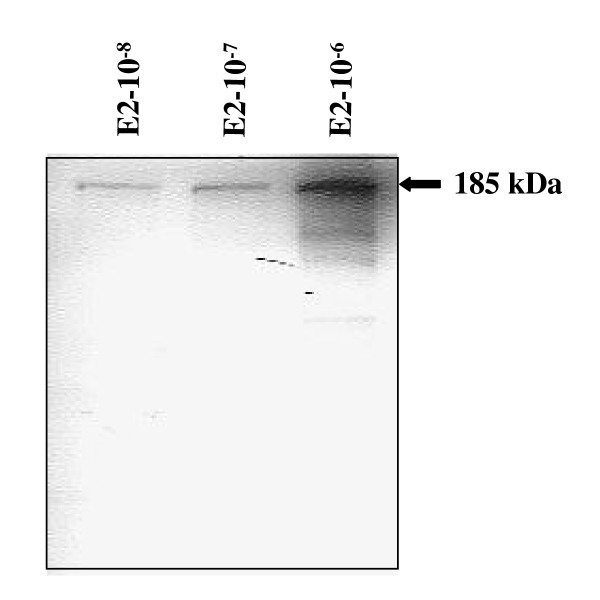
Western blot of plasma from Arctic char exposed to different concentrations of E2 using a polyclonal antibody against Arctic char Vtg.

**Figure 4:**
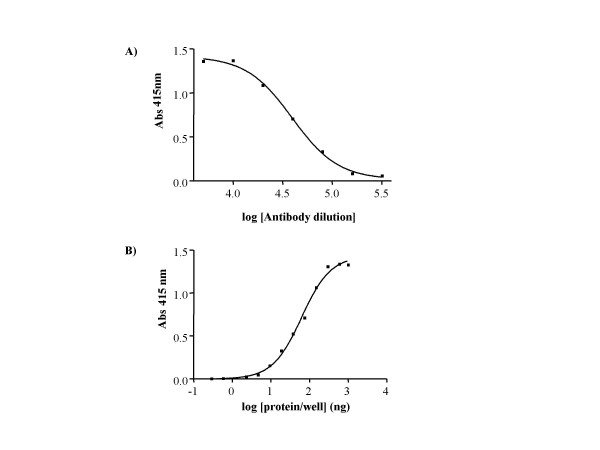
ELISA titration curves. **A) **Titration; A maximum dilution of the antisera was determined to be 10.000×. **B) **Detection limit; An antibody dilution of 1:10.000 was used and the detection limit was determined to 5 ng Vtg/well.

The purified Vtg was used to produce polyclonal Vtg antibodies. The specificity of the polyclonal rabbit antiserum against Arctic char Vtg was determined using western blot analysis. A single band with a molecular mass of 185 kDa was detected only in the plasma of sexually mature females or E2 exposed fish (Fig. 2). To determine if the antibodies could be used quantitatively, plasma from E2 injected fish was separated on SDS-PAGE and detected by western blot analysis. The western blot displayed an increase in plasma Vtg from fish injected with increasing E2 concentrations, further confirming the specificity of the antibodies (Fig. 3). In order to develop an ELISA, the antibodies were tested both at increasing concentrations of antibodies with fixed antigen concentrations and at fixed concentrations of antibodies with increasing concentrations of antigen. The results show that the produced antisera have a high titer allowing dilution up to 10.000 fold without increasing the detection limit (Fig. 4). From these experiments the detection limit of the ELISA was determined to be 5 ng Vtg/well.

Screening of the Arctic char hepatic cDNA library revealed several positive clones. The longest clones were selected and sequenced to completion (clone 1 and clone 3). Sequencing of clone 1 and clone 3 revealed that the Arctic char Vtg mRNA displayed high homology to rainbow trout Vtg mRNA, both at the nucleotide level (89% and 83% respectively) and at the protein level (85% and 82% respectively). Clone 1 and clone 3 showed high similarity (94% on both nucleotide and protein level). In addition, clone 1 was found to contain a second polyadenlyation site and a 116 bases longer 3'UTR. Even though no full-length clones were obtained, these features imply that the clones are products of different genes.

**Figure 5:**
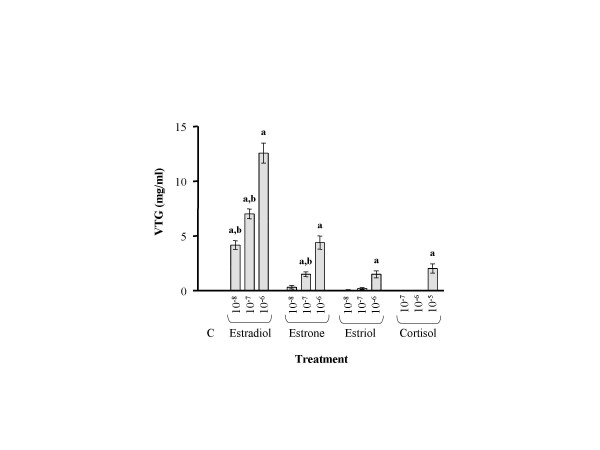
Plasma Vtg concentrations in fish exposed to estrogens and cortisol. Control fish (C) were injected *i.p*. with peanutoil. All values are presented as a mean of 10 fish ± SEM. **a **denotes P < 0.05 when compared with control and **b **denotes P < 0.05 when compared to the highest concentration of each substance.

**Figure 6:**
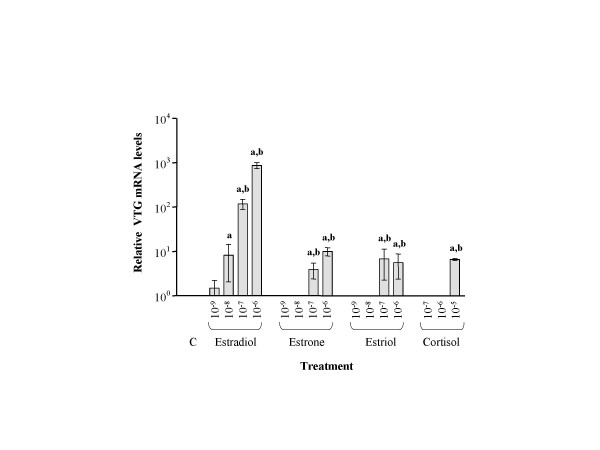
Relative Vtg mRNA levels in fish subjected to *i.p*. administration of estrogens and cortisol. Each bar represents a mean value of three fish ± SEM. Significant differences are marked with **a **and **b**. **a **denotes P < 0.05 when compared with control (C) and **b **denotes P < 0.05 when compared to the highest concentration of each substance.

Eight substances were injected into juvenile Arctic char to determine their potency at inducing Vtg synthesis. ELISA analysis of plasma revealed that only the three estrogens and F induced Vtg synthesis (Fig. 5). The most potent estrogen, E2, was found to be 3 times more effective at inducing Vtg synthesis than estrone and 7 times more potent than the weakest estrogen, estriol. All estrogens induced a dose dependent induction of Vtg. The ability of F to induce Vtg was approximately 70 times lower than E2 and was only observed at the highest dose. Slot blot analysis of Vtg mRNA levels revealed a dose dependent induction corresponding to the induction pattern observed with the ELISA. E2 was the strongest inducer, with both estrone and estriol being weaker but equally potent inducers of Vtg mRNA (Fig. 6). In agreement with the ELISA determinations, F induced Vtg mRNA only at the highest dose. None of the other substances tested displayed any effects on Vtg mRNA.

**Figure 7:**
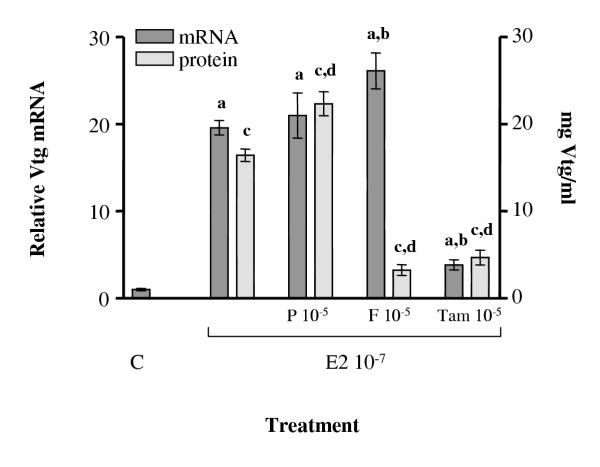
Vtg mRNA and protein levels following co-injection of E2 and P, F and tamoxifen. The dark bar indicates the relative hepatic Vtg mRNA levels while the light bars displays Vtg protein levels present in the plasma. All bars represent a mean value from 5 fish ± SEM. **a **denotes P < 0.05 when compared with control and **b **denotes P < 0.05 when compared to Vtg protein levels in E2 induced fish. **c **denotes P < 0.05 when compared with control (C) and **d **denotes P < 0.05 when compared to Vtg mRNA levels in E2 induced fish.

**Figure 8:**
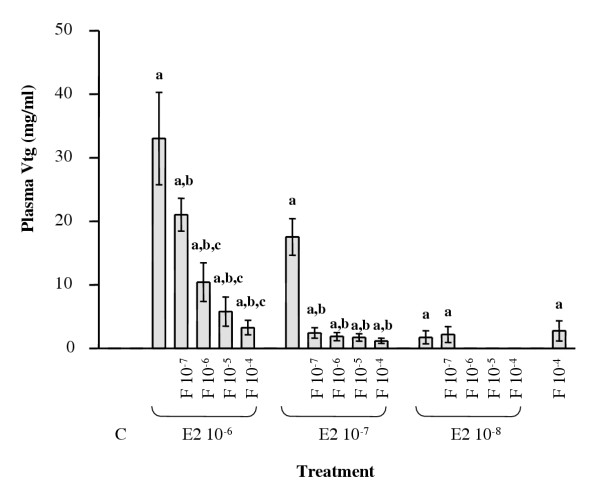
Dose dependent effects of F and E2 on plasma Vtg levels in juvenile Arctic char. All values are presented as a mean value of 5 fish ± SEM. **a **denotes P < 0.05 when compared with control (C). **b **denotes P < 0.05 when compared with each E2 concentrations positive control. **c **denotes P < 0.05 when compared with each E2 + F 10^-7 ^control.

**Figure 9:**
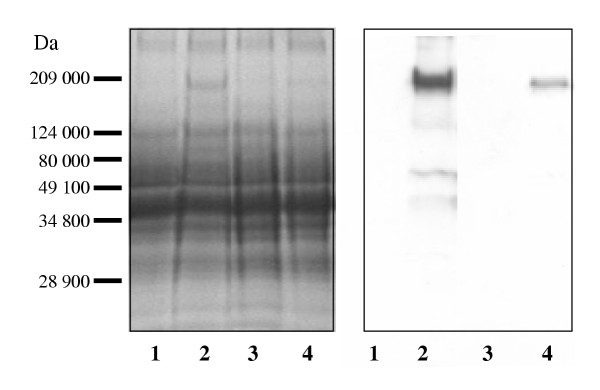
Plasma proteins, 20 μg per lane, from Arctic char treated with 17-β-estradiol (E2, 10^-7 ^M) or/and cortisol (F, 10^-5 ^M) separated on 8% SDS-PAGE. Coomassie-stained gel and corresponding Western blot using a polyclonal antibody against Arctic char vitellogenin. Lane 1: control, lane 2: E2, lane 3: F, lane 4: E2 + F. Molecular weight (Da) are shown to the left.

Arctic char were co-injected with E2 and F, P or tamoxifen in order to determine if other compounds could inhibit Vtg production. Plasma hormone determinations were performed on all groups of fish and the mean plasma levels of E2 and F are shown in table 1. The known antiestrogen tamoxifen was used as a control substance and was found to inhibit the E2 dependent upregulation of both Vtg mRNA and protein levels (Fig. 7). However, while P did not affect the E2 dependent Vtg induction, F co-injection resulted in lowered Vtg protein levels without affecting the Vtg mRNA levels. A second experiment was therefore performed to determine the dose-response effect of co-injection of F with the three different estrogens. ELISA analysis of plasma from co-injected fish reveled dose-dependent inhibition of estrogen induced Vtg levels in plasma (Fig. 8). Western blot of plasma proteins from fish treated with a combination of E2 and F confirmed that F was able to decrease the level of Vtg that are expected in the plasma from an E2-injected fish (Fig. 9).

**Table 1:** Plasma levels of E2 and F following intraperitoneal injections.

**Treatment**	Plasma levels*
Cortisol control	nd
Cortisol 10^-7 ^M	42.3 ± 6.4
Cortisol 10^-6 ^M	132.1 ± 51.8
Cortisol 10^-5 ^M	2593 ± 697
Cortisol 10^-4 ^M	15015 ± 3051
17β-estradiol control	nd
17β-estradiol 10^-8 ^M	10.1 ± 2.8
17β-estradiol 10^-7 ^M	76.7 ± 12.6
17β-estradiol 10^-6^ M	687 ± 75

* The plasma levels are presented as mean (ng/ml) ± S.E.

nd: non detectable levels, below detection limit

**Figure 10:**
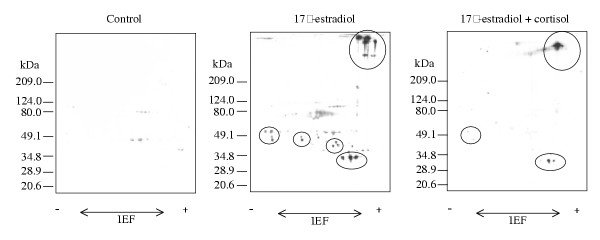
Immunoblots of Arctic char plasma proteins from control, E2- (10^-7 ^M), and E2 + F- (10^-7 ^M and 10^-5 ^M) treated fish separated by two-dimensional electrophoresis. 40 μg total protein was separated by isoelectric focusing in the first dimension using a pH gradient 4–7, followed by SDS-PAGE using 8–18% acrylamide gradient. Polyclonal anti-Arctic char vitellogenin was used. Figure show a part of the PVDF-membrane, spots recognized by the vitellogenin antibody are circled.

The polyclonal antibody directed against a 185 kDa Vtg recognized several high and low molecular weight spots of Vtg and Vtg-derivatives as shown by 2D-PAGE analysis (Fig. 10). Since Vtg is transported in the plasma as a dimmer it migrates as a large complex on 2D-PAGE. There is less of both high and low molecular Vtg-isoforms in the plasma from co-injected fish compared to E2 injected fish.

## Discussion

In this study Arctic char Vtg was purified and polyclonal antibodies was made in order to use Vtg protein determinations as a marker of F effects on egg yolk formation. The purification was performed following the procedure outlined by Silversand and Haux [24]. The chromatographic profile of the purified Arctic char Vtg displayed large similarities when compared to turbot (*Schophthalmus maximus*) [24]. Elution of the protein was obtained at a Cl^- ^concentration of 0.37 M, a value comparable to those earlier reported [29]. The purified Vtg was used to obtain polyclonal antisera from rabbits. The antisera displayed a high specificity for the 185 kDa Vtg, and also recognized Vtg dimers and derivatives as observed by 2D PAGE. Vtg was only detected in females or E2 exposed juvenile Arctic char. It has been found that teleost Vtg, even though highly conserved, may differ in size between 120 – 300 kDa, and are present in the blood plasma mainly as a 300 – 600 kDa dimer [30]. It was also found that the E2 induced Vtg production was dose dependent, as described earlier in many species [31-33].

ELISA procedures have been developed for Vtg from many teleost species [34,35]. This method requires a high specificity of the antibody and a low inter-assay variability. During the evaluation of the antibodies it was found that the antisera contained a high titer of specific Vtg antibodies giving the ELISA a low detection limit of 5 ng Vtg. Low intra and inter assay variability (3%, data not shown) was observed.

Eight substances were tested for their ability to induce Vtg production in Arctic char. It has earlier been shown that Vtg production in teleost fish is under dose dependent estrogenic control [16] and this was also evident in the Arctic char. Presence of Vtg in plasma was detected by the ELISA procedure revealing that Vtg protein was only present in fish exposed to the three estrogens and F. E2 was found to be the most potent estrogen, followed by estrone and estriol. Estriol was the weakest inducer of Vtg synthesis both at mRNA and protein level. These results are in accordance with earlier studies on different species, including human, mouse and rainbow trout [36,37].

The results reported here demonstrate that cortisol acts as a partial antagonist on Vtg expression. The plasma levels of E2 and F following hormone injections showed that the resulting plasma levels covered the range normally observed for Arctic char and other salmonids [38,39]. Exposure of Arctic char to high F levels (10^-5 ^M) resulted in elevated plasma Vtg levels. While F alone induced a low level of Vtg mRNA expression the co exposure of Arctic char to estrogens and F resulted in a reduction in circulating Vtg levels while the Vtg mRNA levels were not affected. These results suggest that F acts at a post-transcriptional level in Arctic char. Our results are in contrast to earlier *in vitro *studies that indicate that F can down regulate Vtg mRNA levels in rainbow trout hepatocytes [18,40], but are supported by a study on *Xenopus *that showed F upregulation of hepatic Vtg production [41]. In *Xenopus *it was suggested that the C/EBPβ-like protein is involved in upregulation of Vtg by increasing the ER levels [41].

Reduced binding of E2 to ER has been observed following F exposure in rainbow trout liver [17]. F has been suggested to interfere with ER transcription by destabilizing ER mRNA, thereby decreasing the mRNA half-life. ER and the glucocorticoid receptor (GR) interact in the liver through C/EBPβ-like protein, and it has been suggested that GR suppress C/EBPβ-like protein binding to the rainbow trout ER promoter, thereby reducing the ER expression [40]. It is known that stress factors are species specific and it cannot be ruled out at the present time that the differences observed between rainbow trout and Arctic char are due to such species differences. However, it should be noted that the earlier studies were conducted on *in vitro *systems as opposed to the whole animal model used in the present study, and that no determination of circulating Vtg levels was performed in the previous studies.

Adding to the complexity of F involvement in reproduction we have recently shown that F potentiates the E2 mediated expression of eggshell protein in Arctic char [38]. F is upregulated during final oocyte maturation and spawning in teleost fish [42]. Thus, it is conceivable that the increase in circulating F levels in maturing female fish is involved in the regulation of eggshell proteins. However, the present results indicate that this involvement is limited to the eggshell proteins as the circulating Vtg levels are reduced under the same conditions.

In the present study the main effect of F was observed at the circulating Vtg level. We hypothesize that the co-treatment of Arctic char with glucocorticoids and estrogens results in upregulation of both stress induced systems, such as metallothionein (MT), and estrogen responsive genes, such as eggshell proteins and vitellogenin. MT has been shown to be upregulated by cortisol [43] in rainbow trout primary cultures and has a main function to sequester zinc [44]. The involvement of MT in fish reproduction has been shown previously for rainbow trout and Arctic char [38,39]. In both species MT is upregulated towards the end of vitellogenesis [38,39] and is believed to sequester Zn from the liver in order to control the Zn homeostasis once vitellogenesis is over [45]. It has also been shown that E2 functions as an antagonist of MT induction in both rainbow trout [28] and Arctic char [46] further supporting the involvement of MT in reproduction. If Vtg requires Zn for proper tertiary folding, then upregulation of MT by cortisol could lead to a redistribution of Zn from Vtg to MT with degradation of Vtg as a consequence. As egg shell proteins do not use Zn as a structural motif the upregulation of MT would not have the same effect on eggshell proteins. This is in part confirmed by our previous study showing that F potentiates estrogenic induction of eggshell proteins. Further studies are underway to determine the cause of the reduction in circulating Vtg levels.
